# The Home Hospitals Association

**Published:** 1893-03-25

**Authors:** 


### The Home Hospitals Association.

The 15th annual meeting of the above association was held at Fitzroy House, Fitzroy Square, on Tuesday, March 21st, at four p.m. The chair was taken by Dr. Hare. The report for the year 1892 was read by the Secretary (Mr. Almond-Hind), showing that the work of the hospital had been carried on in a most satisfactory manner. In April the accounts of the Nurses' Home and the Hospital were amalgamated, an arrangement to be adhered to in the future. The Lady Superintendent, Miss Judkins, had resigned, and been succeeded by Miss Pearson, who had been Assistant Superintendent since 1891; Miss Monk was now her assistant. With the exception of one nurse, who left to be married, the nursing staff remains the same as last year. The Nurses' Home has proved a decided success; there are now 13 nurses on the staff, all of whom have been kept busy during the year. The condition of the finances is satisfactory. The total receipts for the year amounted to £5,103 16s. 4d., and the expenditure to £5,015 9s. 2d., giving a balance in favour of the hospital of £88 7s. 2d.

For the year 1892 there had been 412 applications for admission, and 302 admissions. Sixty-six medical men had had patients in the hospital during the year, and all expressed themselves thoroughly satisfied with the attention their patients received from the nursing staff, the patients, without exception, endorsing that opinion.

The Chairman, in moving a resolution that the report be adopted, printed, and circulated, said that the small number of persons present bore witness to the excellence of the management of the hospital, inasmuch as it intimated that there was no fault to be found with the administration. Speaking of the need that existed for such hospitals for the middle classes, Dr. Hare said it was hoped in time to extend the work of the hospital by the establishment of convalescent homes. There were more applications than the houses could accommodate, and patients received there went away more than satisfied.

Mr. Christopher Heath, in seconding the resolution, said he preferred to have his patients at the home than elsewhere, and fully corroborated the statements made as to the efficiency of the nursing. Patients were always sorry to leave.

The members of the executive board having been re-elected, Dr. Fenton proposed a vote of thanks to the members of the medical board of reference and executive staff, which was cordially seconded and passed. He should like to refer to a compliment on the appearance of the nurses just made by a gentleman sitting near him, who remarked that if he had been a woman he should have wished to be a nurse, as judging from those present they seemed to possess the secret of perpetual youth and happiness.

Dr. Tom Robinson offered a suggestion that the electric light should be adopted in the hospital, as the heat of some of the upper rooms had been complained of, but the chairman of the board of management, while thanking him for the proposal, feared the funds would not permit such a plan to be carried out at present.

The formal proceedings terminated with a cordial vote of thanks to the chairman, proposed by Sir Richard Quain. Tea and coffee were afterwards served to the visitors, and the pleasant and kindly tone pervading the Home was specially noticeable in this friendly gathering. The doctors not only warmly appreciate the good nursing at Fitzroy House, but are evidently anxious to give it full acknowledgment. The benefits conferred on the community at large by Pay Hospitals are too obvious to need detailing at present. But to the pioneers who first opened Fitzroy House as a home hospital the thanks of the great middle class now largely supporting it owe a large debt of gratitude.

